# Gas

**Published:** 1881-05

**Authors:** William D. Kempton


BY WILLIAM D. KEMPTON, M. D., D. D. S.

The gas in question is not $N_2O$, neither is it an anæsthetic, although it sometimes causes drowsiness, but more often is irritating and painful. I refer to the superfluous wind generated in the various dental sooieties by members who seem to have fallen in love with the sound of their own voices; some of whom waste so much time in preliminary remarks that when they do get to the point their hearers, having grown weary and impatient are not paying attention, and so it escapes them.

There are others who, after some one has stated a point briefly and concisely, will arise and say, "I heartily indorse what Dr. So and So has just said, it is very important, &c.," and continue in that strain, reiterating what others have said, occupying the time of the society, and do not advance a single idea.

These persons seem to forget that many of their auditors who have come from a distance, and are generally men of an inquiring mind, come for the purpose of adding to their stock of knowledge, and perhaps to contribute their mite to the general fund, and, if they are to be bored by being compelled to listen to one or more inveterate gas bags, will deliberate a long time before going to the trouble and expense of a second visit.

Therefore, if one has anything to communicate let him say it, briefly and to the point. If he has nothing to add to the remarks already made let him keep still, for "life is short."

